# 2-Oxo-4-phenyl-1,2,5,6-tetra­hydro­benzo[*h*]quinoline-3-carbonitrile

**DOI:** 10.1107/S1600536811033873

**Published:** 2011-08-27

**Authors:** Abdullah M. Asiri, Hassan M. Faidallah, Abdulrahman O. Al-Youbi, Khalid A. Alamry, Seik Weng Ng

**Affiliations:** a Chemistry Department, Faculty of Science, King Abdulaziz University, PO Box 80203 Jeddah, Saudi Arabia; b Department of Chemistry, University of Malaya, 50603 Kuala Lumpur, Malaysia

## Abstract

In the mol­ecule of the title compound, C_20_H_14_N_2_O, the tetra­hydro­benzo[*h*]quinoline fused-ring system is buckled owing to the ethyl­ene –CH_2_CH_2_– fragment, the benzene ring and the pyridine ring being twisted by 19.7 (1)°. The 4-substituted aromatic ring is bent away from the pyridine ring by 62.9 (1)° in order to avoid crowding the cyanide substituent. In the crystal, two mol­ecules are linked by a pair of N—H⋯O hydrogen bonds to form a centrosymmetric dimer.

## Related literature

The title compound belongs to a series of cyano-pyridino­nes that have been evaluated for their anti­cancer properties, see: Rostom *et al.* (2011 ▶).
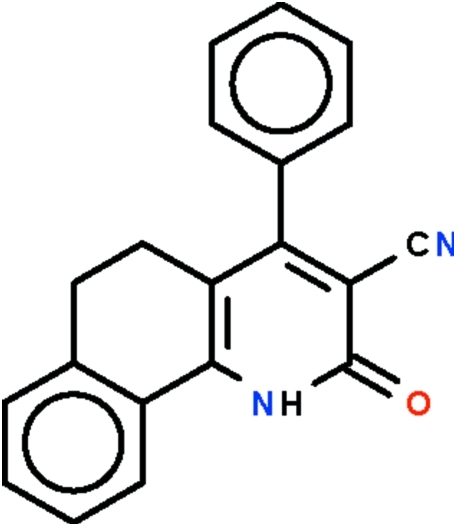


## Experimental

### 

#### Crystal data

C_20_H_14_N_2_O
*M* *_r_* = 298.33
Triclinic, 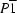

*a* = 7.4075 (5) Å
*b* = 9.7204 (4) Å
*c* = 10.7358 (6) Å
α = 77.001 (4)°
β = 74.348 (6)°
γ = 81.674 (5)°
*V* = 722.36 (7) Å^3^
*Z* = 2
Cu *K*α radiation
μ = 0.68 mm^−1^
*T* = 100 K
0.35 × 0.30 × 0.25 mm

#### Data collection

Agilent SuperNova Dual diffractometer with Atlas detector
Absorption correction: multi-scan (*CrysAlis PRO*; Agilent, 2010 ▶) *T*_min_ = 0.797, *T*_max_ = 0.848
4086 measured reflections
2785 independent reflections
2576 reflections with *I* > 2σ(*I*)
*R*_int_ = 0.015

#### Refinement

*R*[*F* ^2^ > 2σ(*F* ^2^)] = 0.037
*wR*(*F* ^2^) = 0.106
*S* = 1.03
2785 reflections
212 parameters
H atoms treated by a mixture of independent and constrained refinement
Δρ_max_ = 0.24 e Å^−3^
Δρ_min_ = −0.27 e Å^−3^

### 

Data collection: *CrysAlis PRO* (Agilent, 2010 ▶); cell refinement: *CrysAlis PRO*; data reduction: *CrysAlis PRO*; program(s) used to solve structure: *SHELXS97* (Sheldrick, 2008 ▶); program(s) used to refine structure: *SHELXL97* (Sheldrick, 2008 ▶); molecular graphics: *X-SEED* (Barbour, 2001 ▶); software used to prepare material for publication: *publCIF* (Westrip, 2010 ▶).

## Supplementary Material

Crystal structure: contains datablock(s) global, I. DOI: 10.1107/S1600536811033873/xu5290sup1.cif

Structure factors: contains datablock(s) I. DOI: 10.1107/S1600536811033873/xu5290Isup2.hkl

Supplementary material file. DOI: 10.1107/S1600536811033873/xu5290Isup3.cml

Additional supplementary materials:  crystallographic information; 3D view; checkCIF report

## Figures and Tables

**Table 1:** Hydrogen-bond geometry (Å, °)

*D*—H⋯*A*	*D*—H	H⋯*A*	*D*⋯*A*	*D*—H⋯*A*
N1—H1⋯O1^i^	0.97 (2)	1.89 (2)	2.848 (1)	168 (1)

Symmetry code: (i) 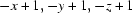.

**Fig. 1.**
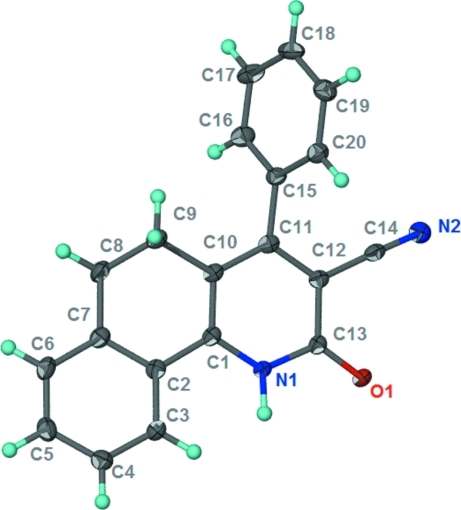
Thermal ellipsoid plot (Barbour, 2001) of C20H14N2O at the 70% probability level; hydrogen atoms are drawn as spheres of arbitrary radius.

## supplementary crystallographic information

### Comment

The compound (Scheme I) belongs to a series of cyano-pyridinones that have been evaluated for their anticancer properties (Rostom *et al.*, 2011). The tetrahydrobenzo[*h*]quinoline fused-ring system is buckled owing to the ethylene –CH_2_CH_2_– fragment, the benzene ring and the pyridine ring being twisted by 19.7 (1)°. The 4-subsituted aromatic ring is bent away from the pyridine ring by 62.9 (1) ° in order to avoid crowding the cyanide substituent (Fig. 1). Two molecules are linked by an N—H···O hydrogen bonds to form a centrosymmetric dimer (Table 1).

### Experimental

A mixture of benzaldehyde (1.06 g, 10 mmol), 1-tetralone (1.46 g, 10 mmol), ethyl cyanoacetate (1.1 g, 10 mmol) and ammonium acetate (6.2 g, 80 mmol) in absolute ethanol (50 ml) was refluxed for 6 h. The reaction mixture was allowed to cool, and the orange precipitate that formed was filtered, washed with water, dried and recrystallized from ethanol; m.p. 585–597 K.

### Refinement

Carbon- and nitrogen-bound H atoms were placed in calculated positions [C—H 0.95 to 0.99 Å, *U*_iso_(H) = 1.2*U*_eq_(C)] and were included in the refinement in the riding model approximation.

The amino H atom was located in a difference Fourier map and was freely refined.

The diffraction data are 94% complete at a 2θ limit of 150° but are 99% complete at 135°.

### Crystal data

**Table:**

C_20_H_14_N_2_O	*Z* = 2
*M**_r_* = 298.33	*F*(000) = 312
Triclinic, *P*1	*D*_x_ = 1.372 Mg m^−^^3^
Hall symbol: -P 1	Cu *K*α radiation, λ = 1.54184 Å
*a* = 7.4075 (5) Å	Cell parameters from 2774 reflections
*b* = 9.7204 (4) Å	θ = 4.4–74.3°
*c* = 10.7358 (6) Å	µ = 0.68 mm^−^^1^
α = 77.001 (4)°	*T* = 100 K
β = 74.348 (6)°	Block, yellow
γ = 81.674 (5)°	0.35 × 0.30 × 0.25 mm
*V* = 722.36 (7) Å^3^	

### Data collection

**Table:**

Agilent SuperNova Dual diffractometer with Atlas detector	2785 independent reflections
Radiation source: SuperNova (Cu) X-ray Source	2576 reflections with *I* > 2σ(*I*)
mirror	*R*_int_ = 0.015
Detector resolution: 10.4041 pixels mm^-1^	θ_max_ = 74.5°, θ_min_ = 4.4°
ω scans	*h* = −9→8
Absorption correction: multi-scan (*CrysAlis PRO*; Agilent, 2010)	*k* = −12→9
*T*_min_ = 0.797, *T*_max_ = 0.848	*l* = −12→13
4086 measured reflections	

### Refinement

**Table:**

Refinement on *F*^2^	Primary atom site location: structure-invariant direct methods
Least-squares matrix: full	Secondary atom site location: difference Fourier map
*R*[*F*^2^ > 2σ(*F*^2^)] = 0.037	Hydrogen site location: inferred from neighbouring sites
*wR*(*F*^2^) = 0.106	H atoms treated by a mixture of independent and constrained refinement
*S* = 1.03	*w* = 1/[σ^2^(*F*_o_^2^) + (0.0667*P*)^2^ + 0.1847*P*] where *P* = (*F*_o_^2^ + 2*F*_c_^2^)/3
2785 reflections	(Δ/σ)_max_ = 0.001
212 parameters	Δρ_max_ = 0.24 e Å^−^^3^
0 restraints	Δρ_min_ = −0.27 e Å^−^^3^

### Special details

**Table:**

Geometry. All e.s.d.'s (except the e.s.d. in the dihedral angle between two l.s. planes) are estimated using the full covariance matrix. The cell e.s.d.'s are taken into account individually in the estimation of e.s.d.'s in distances, angles and torsion angles; correlations between e.s.d.'s in cell parameters are only used when they are defined by crystal symmetry. An approximate (isotropic) treatment of cell e.s.d.'s is used for estimating e.s.d.'s involving l.s. planes.

### Fractional atomic coordinates and isotropic or equivalent isotropic displacement parameters (Å^2^)

**Table:**

	*x*	*y*	*z*	*U*_iso_*/*U*_eq_	
O1	0.36679 (10)	0.61389 (8)	0.41605 (8)	0.0164 (2)	
N1	0.24537 (13)	0.48620 (10)	0.62038 (9)	0.0133 (2)	
N2	0.01407 (14)	0.86078 (10)	0.31963 (10)	0.0196 (2)	
C1	0.10482 (15)	0.45737 (11)	0.73289 (11)	0.0134 (2)	
C2	0.14223 (15)	0.34124 (11)	0.83986 (11)	0.0144 (2)	
C3	0.32488 (15)	0.28586 (12)	0.84832 (11)	0.0163 (2)	
H3	0.4303	0.3258	0.7854	0.020*	
C4	0.35215 (16)	0.17277 (12)	0.94846 (12)	0.0188 (3)	
H4	0.4762	0.1361	0.9545	0.023*	
C5	0.19842 (17)	0.11307 (12)	1.03992 (12)	0.0197 (3)	
H5	0.2173	0.0342	1.1071	0.024*	
C6	0.01712 (16)	0.16880 (12)	1.03297 (11)	0.0186 (3)	
H6	−0.0874	0.1280	1.0963	0.022*	
C7	−0.01372 (15)	0.28330 (12)	0.93487 (11)	0.0157 (2)	
C8	−0.20935 (15)	0.34442 (12)	0.92477 (11)	0.0185 (3)	
H8A	−0.2982	0.3238	1.0126	0.022*	
H8B	−0.2498	0.2995	0.8639	0.022*	
C9	−0.21419 (16)	0.50443 (12)	0.87427 (11)	0.0178 (3)	
H9A	−0.3407	0.5420	0.8612	0.021*	
H9B	−0.1885	0.5508	0.9401	0.021*	
C10	−0.06775 (15)	0.53738 (12)	0.74493 (11)	0.0142 (2)	
C11	−0.09384 (15)	0.64960 (11)	0.64016 (11)	0.0140 (2)	
C12	0.05055 (15)	0.67305 (11)	0.52614 (11)	0.0138 (2)	
C13	0.23126 (15)	0.59235 (11)	0.51369 (11)	0.0132 (2)	
C14	0.02822 (14)	0.77868 (11)	0.41323 (11)	0.0148 (2)	
C15	−0.26822 (15)	0.74948 (12)	0.65116 (11)	0.0146 (2)	
C16	−0.44492 (16)	0.70524 (12)	0.66625 (11)	0.0186 (3)	
H16	−0.4573	0.6088	0.6681	0.022*	
C17	−0.60284 (16)	0.80230 (14)	0.67855 (12)	0.0217 (3)	
H17	−0.7230	0.7722	0.6877	0.026*	
C18	−0.58610 (17)	0.94329 (13)	0.67750 (12)	0.0218 (3)	
H18	−0.6949	1.0088	0.6878	0.026*	
C19	−0.41072 (17)	0.98783 (12)	0.66149 (12)	0.0210 (3)	
H19	−0.3989	1.0843	0.6600	0.025*	
C20	−0.25188 (16)	0.89167 (12)	0.64761 (11)	0.0176 (2)	
H20	−0.1315	0.9228	0.6356	0.021*	
H1	0.372 (2)	0.4387 (17)	0.6103 (16)	0.029 (4)*	

### Atomic displacement parameters (Å^2^)

**Table:**

	*U*^11^	*U*^22^	*U*^33^	*U*^12^	*U*^13^	*U*^23^
O1	0.0117 (4)	0.0184 (4)	0.0152 (4)	0.0000 (3)	0.0004 (3)	−0.0005 (3)
N1	0.0103 (4)	0.0144 (4)	0.0135 (5)	−0.0003 (3)	−0.0016 (3)	−0.0017 (4)
N2	0.0182 (5)	0.0197 (5)	0.0192 (5)	0.0001 (4)	−0.0040 (4)	−0.0019 (4)
C1	0.0123 (5)	0.0149 (5)	0.0134 (5)	−0.0031 (4)	−0.0017 (4)	−0.0042 (4)
C2	0.0154 (5)	0.0148 (5)	0.0131 (5)	−0.0013 (4)	−0.0024 (4)	−0.0046 (4)
C3	0.0142 (5)	0.0179 (5)	0.0147 (5)	−0.0018 (4)	−0.0004 (4)	−0.0026 (4)
C4	0.0175 (5)	0.0194 (6)	0.0182 (6)	0.0011 (4)	−0.0048 (4)	−0.0019 (4)
C5	0.0244 (6)	0.0170 (5)	0.0154 (6)	−0.0011 (4)	−0.0037 (5)	−0.0005 (4)
C6	0.0186 (6)	0.0193 (6)	0.0150 (6)	−0.0047 (4)	0.0012 (4)	−0.0020 (4)
C7	0.0151 (5)	0.0174 (5)	0.0146 (5)	−0.0025 (4)	−0.0017 (4)	−0.0050 (4)
C8	0.0133 (5)	0.0231 (6)	0.0162 (6)	−0.0042 (4)	0.0005 (4)	−0.0009 (4)
C9	0.0143 (5)	0.0217 (6)	0.0143 (6)	0.0008 (4)	0.0002 (4)	−0.0031 (4)
C10	0.0122 (5)	0.0161 (5)	0.0145 (5)	−0.0018 (4)	−0.0016 (4)	−0.0049 (4)
C11	0.0122 (5)	0.0153 (5)	0.0162 (5)	−0.0019 (4)	−0.0034 (4)	−0.0061 (4)
C12	0.0120 (5)	0.0145 (5)	0.0152 (5)	−0.0009 (4)	−0.0033 (4)	−0.0037 (4)
C13	0.0127 (5)	0.0136 (5)	0.0132 (5)	−0.0026 (4)	−0.0020 (4)	−0.0031 (4)
C14	0.0103 (5)	0.0154 (5)	0.0186 (6)	−0.0013 (4)	−0.0013 (4)	−0.0056 (5)
C15	0.0133 (5)	0.0178 (5)	0.0117 (5)	0.0013 (4)	−0.0022 (4)	−0.0038 (4)
C16	0.0168 (6)	0.0204 (6)	0.0197 (6)	−0.0006 (4)	−0.0038 (4)	−0.0074 (5)
C17	0.0126 (5)	0.0319 (7)	0.0219 (6)	0.0008 (5)	−0.0046 (4)	−0.0091 (5)
C18	0.0176 (6)	0.0258 (6)	0.0192 (6)	0.0084 (5)	−0.0040 (4)	−0.0060 (5)
C19	0.0235 (6)	0.0176 (6)	0.0195 (6)	0.0032 (5)	−0.0033 (5)	−0.0044 (4)
C20	0.0156 (5)	0.0190 (6)	0.0171 (6)	−0.0009 (4)	−0.0024 (4)	−0.0039 (4)

### Geometric parameters (Å, °)

**Table:**

O1—C13	1.2429 (13)	C8—H8B	0.9900
N1—C1	1.3694 (14)	C9—C10	1.5150 (15)
N1—C13	1.3745 (14)	C9—H9A	0.9900
N1—H1	0.970 (17)	C9—H9B	0.9900
N2—C14	1.1530 (15)	C10—C11	1.4130 (16)
C1—C10	1.3870 (15)	C11—C12	1.3904 (15)
C1—C2	1.4723 (15)	C11—C15	1.4938 (15)
C2—C3	1.4007 (16)	C12—C14	1.4318 (15)
C2—C7	1.4094 (15)	C12—C13	1.4393 (15)
C3—C4	1.3879 (16)	C15—C16	1.3934 (16)
C3—H3	0.9500	C15—C20	1.3959 (16)
C4—C5	1.3890 (16)	C16—C17	1.3889 (16)
C4—H4	0.9500	C16—H16	0.9500
C5—C6	1.3884 (17)	C17—C18	1.3911 (18)
C5—H5	0.9500	C17—H17	0.9500
C6—C7	1.3888 (16)	C18—C19	1.3834 (18)
C6—H6	0.9500	C18—H18	0.9500
C7—C8	1.5071 (16)	C19—C20	1.3892 (16)
C8—C9	1.5262 (16)	C19—H19	0.9500
C8—H8A	0.9900	C20—H20	0.9500
			
C1—N1—C13	124.83 (9)	C8—C9—H9B	109.7
C1—N1—H1	123.1 (9)	H9A—C9—H9B	108.2
C13—N1—H1	111.8 (9)	C1—C10—C11	118.74 (10)
N1—C1—C10	120.15 (10)	C1—C10—C9	117.52 (10)
N1—C1—C2	118.52 (9)	C11—C10—C9	123.63 (10)
C10—C1—C2	121.33 (10)	C12—C11—C10	119.34 (10)
C3—C2—C7	119.71 (10)	C12—C11—C15	118.71 (10)
C3—C2—C1	122.50 (10)	C10—C11—C15	121.85 (10)
C7—C2—C1	117.78 (10)	C11—C12—C14	121.99 (10)
C4—C3—C2	120.15 (10)	C11—C12—C13	122.24 (10)
C4—C3—H3	119.9	C14—C12—C13	115.77 (9)
C2—C3—H3	119.9	O1—C13—N1	121.03 (9)
C3—C4—C5	120.14 (11)	O1—C13—C12	124.38 (10)
C3—C4—H4	119.9	N1—C13—C12	114.59 (9)
C5—C4—H4	119.9	N2—C14—C12	177.69 (12)
C6—C5—C4	119.91 (11)	C16—C15—C20	119.43 (10)
C6—C5—H5	120.0	C16—C15—C11	122.37 (10)
C4—C5—H5	120.0	C20—C15—C11	118.19 (10)
C5—C6—C7	121.00 (11)	C17—C16—C15	119.90 (11)
C5—C6—H6	119.5	C17—C16—H16	120.1
C7—C6—H6	119.5	C15—C16—H16	120.1
C6—C7—C2	119.05 (10)	C16—C17—C18	120.39 (11)
C6—C7—C8	121.83 (10)	C16—C17—H17	119.8
C2—C7—C8	119.09 (10)	C18—C17—H17	119.8
C7—C8—C9	110.56 (9)	C19—C18—C17	119.86 (10)
C7—C8—H8A	109.5	C19—C18—H18	120.1
C9—C8—H8A	109.5	C17—C18—H18	120.1
C7—C8—H8B	109.5	C18—C19—C20	120.05 (11)
C9—C8—H8B	109.5	C18—C19—H19	120.0
H8A—C8—H8B	108.1	C20—C19—H19	120.0
C10—C9—C8	109.84 (9)	C19—C20—C15	120.35 (11)
C10—C9—H9A	109.7	C19—C20—H20	119.8
C8—C9—H9A	109.7	C15—C20—H20	119.8
C10—C9—H9B	109.7		
			
C13—N1—C1—C10	0.43 (17)	C1—C10—C11—C12	2.66 (16)
C13—N1—C1—C2	−179.06 (9)	C9—C10—C11—C12	178.76 (10)
N1—C1—C2—C3	18.21 (16)	C1—C10—C11—C15	−173.72 (10)
C10—C1—C2—C3	−161.28 (11)	C9—C10—C11—C15	2.38 (17)
N1—C1—C2—C7	−160.55 (10)	C10—C11—C12—C14	175.67 (10)
C10—C1—C2—C7	19.96 (15)	C15—C11—C12—C14	−7.84 (16)
C7—C2—C3—C4	1.16 (17)	C10—C11—C12—C13	−3.94 (17)
C1—C2—C3—C4	−177.57 (10)	C15—C11—C12—C13	172.55 (9)
C2—C3—C4—C5	0.66 (17)	C1—N1—C13—O1	178.43 (10)
C3—C4—C5—C6	−1.51 (18)	C1—N1—C13—C12	−1.51 (15)
C4—C5—C6—C7	0.53 (18)	C11—C12—C13—O1	−176.65 (10)
C5—C6—C7—C2	1.27 (17)	C14—C12—C13—O1	3.71 (16)
C5—C6—C7—C8	179.37 (11)	C11—C12—C13—N1	3.27 (16)
C3—C2—C7—C6	−2.10 (16)	C14—C12—C13—N1	−176.36 (9)
C1—C2—C7—C6	176.69 (10)	C12—C11—C15—C16	119.29 (12)
C3—C2—C7—C8	179.74 (10)	C10—C11—C15—C16	−64.31 (15)
C1—C2—C7—C8	−1.47 (15)	C12—C11—C15—C20	−61.49 (14)
C6—C7—C8—C9	146.11 (11)	C10—C11—C15—C20	114.91 (12)
C2—C7—C8—C9	−35.79 (14)	C20—C15—C16—C17	−0.47 (17)
C7—C8—C9—C10	54.80 (13)	C11—C15—C16—C17	178.75 (10)
N1—C1—C10—C11	−0.94 (16)	C15—C16—C17—C18	−0.78 (18)
C2—C1—C10—C11	178.54 (9)	C16—C17—C18—C19	1.29 (18)
N1—C1—C10—C9	−177.28 (10)	C17—C18—C19—C20	−0.54 (18)
C2—C1—C10—C9	2.20 (16)	C18—C19—C20—C15	−0.70 (18)
C8—C9—C10—C1	−39.57 (14)	C16—C15—C20—C19	1.21 (17)
C8—C9—C10—C11	144.29 (11)	C11—C15—C20—C19	−178.04 (10)

### Hydrogen-bond geometry (Å, °)

**Table:**

*D*—H···*A*	*D*—H	H···*A*	*D*···*A*	*D*—H···*A*
N1—H1···O1^i^	0.97 (2)	1.89 (2)	2.848 (1)	168 (1)

Symmetry codes: (i) −*x*+1, −*y*+1, −*z*+1.
